# X- ray crystallography reveals the mechanism of SARS-CoV-2 PLpro dimerization mediated by a DNA-encoded library screening hit

**DOI:** 10.1063/4.0000814

**Published:** 2025-10-27

**Authors:** Orville Pemberton, Amanda M Nevins, Thomas E Frederick, Emily Nicholl, Myron Srikumaran, Jun Chen, Alla Korepanova, Vincent Stoll, Andrew Petros, Sujatha Gopalakrishnan, Justin Dietrich, Liliam Rios Cordero, David J Hardee, Teresa I Ng, Chaohong Sun

**Affiliations:** AbbVie, North Chicago, IL, USA

## Abstract

The COVID-19 pandemic caused by SARS-CoV-2 has devastated global health, revealing an urgent need for novel therapeutics. The papain-like protease (PLpro) is one of two proteases encoded by SARS-CoV-2, representing an attractive drug target due to its dual roles in viral replication and host immune suppression. We employed a DNA-encoded library (DEL) screen to reveal starting points for our PLpro hit discovery campaign. These efforts led to the identification of compound 1, a diarylmethanol-containing substructure with a unique binding mode that induces PLpro dimerization. Compound 1 demonstrates potent activities in both a biochemical ubiquitin-rhodamine and antiviral HeLa-ACE2 cell assays. An X- ray co-crystal structure of compound 1 bound to PLpro solved to 2.0 Å showed that two molecules of compound 1 glues two monomers of PLpro together via binding to the BL2 groove of one monomer and the Ubl/thumb domain of the other. Several molecular interactions were observed between compound 1 and PLpro including hydrophobic interactions and several hydrogen-bonds across both monomers. The molecular glue-like properties of compound 1 on PLpro were further validated in solution with analytical SEC and protein-detect 2D-NMR. Subsequent rounds of SAR led to compound 2, which has comparable biochemical and antiviral activities and demonstrated the same dimerization mechanism of PLpro as seen in a 1.8 Å X-ray co-crystal structure. In summary, we have identified a new series of PLpro inhibitors with a novel mechanism of SARS-CoV-2 inhibition, providing a promising start for the discovery of antivirals for treating COVID-19.

